# Global quantitative proteome analysis of a multi-resistant *Klebsiella pneumoniae* strain

**DOI:** 10.3389/fmicb.2025.1528869

**Published:** 2025-05-19

**Authors:** Marie-Sofie Illenseher, Christian Hentschker, Manuela Gesell Salazar, Larissa Milena Busch, Lisa Zierke, Alexander Reder, Stephan Michalik, Uwe Völker, Sven Hammerschmidt, Kristin Surmann

**Affiliations:** ^1^Department of Molecular Genetics and Infection Biology, Center for Functional Genomics of Microbes, Interfaculty Institute of Genetics and Functional Genomics, University of Greifswald, Greifswald, Germany; ^2^Department of Functional Genomics, Interfaculty Institute of Genetics and Functional Genomics, University Medicine Greifswald, Greifswald, Germany

**Keywords:** *Klebsiella pneumoniae*, global quantitative proteomics, heat stress, oxidative stress, outer membrane proteins, lipoproteins

## Abstract

*Klebsiella pneumoniae* is a critical nosocomial pathogen with a rising incidence in antibiotic resistance worldwide. Multidrug-resistant strains of *K. pneumoniae* pose a major health threat, particularly to immunosuppressed and elderly patients. To date, no effective vaccine formulations have been developed. Therefore, the creation of novel therapeutic and preventive treatments is of key importance. Proteins play a central role in host-pathogen interactions, and identifying and characterizing conserved, organism-specific proteins is essential for the creation of novel vaccine formulations. Capsule-deficient *K. pneumoniae* ATCC BAA-2146Δ*wza* was cultured in minimal medium and exposed to two physiological stress factors to profile its cellular proteome and exoproteome and its adaptation to infection mimicking conditions by mass spectrometry. More than 2,800 proteins covering 54% of the entire annotated proteome, were profiled in untreated controls and after imposition of heat or oxidative stress. The proteomic data revealed that some outer membrane proteins (OMPs), including LPP and other virulence factors involved in iron acquisition and cell wall remodeling, were more abundant in the exoproteome than in the cellular proteome. Approximately one-third of the assigned OMPs were lipoproteins, i.e., proteins that represent important fitness factors and might be immunogenic due to their structure and position in the bacterial cell membrane. In particular, the lipoproteins Pal and SlyB showed the highest abundance among the OM lipoproteins in the intracellular proteome and exoproteome in all tested conditions. These findings suggest that these proteins may be promising candidates for further immunoproteomic analyses to assess their immunogenicity and their role in *K. pneumoniae* pathogenicity.

## 1 Introduction

Together with *Staphylococcus aureus* (*S. aureus*), *Escherichia coli* (*E. coli*), *Streptococcus pneumoniae*, and *Pseudomonas aeruginosa*, *Klebsiella pneumoniae* (*K. pneumoniae*) belongs to the five major pathogens responsible for half of the global deaths caused by bacterial infections (GBD 2019 Antimicrobial Resistance Collaborators, 2022). *K. pneumoniae* is a Gram-negative, opportunistic pathogen that initially colonizes the mucous membranes of the nasopharynx and gastrointestinal tract as a commensal (Bagley, 1985; Paczosa and Mecsas, 2016). However, *K. pneumoniae* can migrate to surrounding tissues or the bloodstream under conditions of a weakened host immune system, where it can cause life-threatening diseases (Boucher et al., 2009; Paczosa and Mecsas, 2016). These diseases include pneumonia, urinary tract infections, and bacteremia, posing a major health risk for newborns, immunosuppressed, and elderly patients (Finland et al., 1959; Selden et al., 1971; Boucher et al., 2009; Richelsen et al., 2020). The emergence of hypervirulent and multidrug-resistant strains of *K. pneumoniae* is a growing concern in both community-acquired and nosocomial infections. This is driven by the overuse and misuse of antibiotics, as well as the lack of innovation in the antibiotic discovery over recent decades. Some multi-resistant *K. pneumoniae* strains produce extended spectrum beta-lactamases (ESBLs) that hydrolyze and inactivate β-lactams, including carbapenems (Pitout and Laupland, 2008; Ballén et al., 2021; Ahmed et al., 2022). Carbapenems are standard antimicrobial treatments for infections, making the increasing prevalence of multi-resistant, carbapenemase-producing *K. pneumoniae* strains a major threat to global healthcare systems (Grundmann et al., 2017; Ding et al., 2023). According to a global report by the World Health Organization (WHO) on antibiotic resistance surveillance, up to 50% of *K. pneumoniae* isolates from severe nosocomial infections showed resistance to third-generation cephalosporins and carbapenems^1^ (WHO, Antimicrobial resistance: global report on surveillance, World Health Organization, 2014). Given these challenges, it is crucial to develop innovative strategies to combat *K. pneumoniae* infections, particularly those caused by multi-resistant strains, through infection prevention methods such as the development of new vaccines (Bassetti et al., 2015; Assoni et al., 2021). Despite ongoing efforts, effective vaccines against *K. pneumoniae* infections remain unavailable, highlighting the need for alternative approaches. The extensive strain variability poses a challenge for broadly protective formulations, emphasizing the importance of identifying conserved targets beyond capsular polysaccharides. In this context, protein-based vaccine candidates offer a promising solution. Global proteomic profiling of multi-resistant *K. pneumoniae* strains could facilitate the identification of such targets, contributing to the development of effective prevention and treatment strategies.

During infection, pathogens must overcome several barriers of the host, adapt to host defense mechanisms, and potentially invade host cells and tissues (Oelschlaeger and Tall, 1997; Hsu et al., 2015; Gorrie et al., 2017). These challenges include oxidative stress, limited access to essential trace elements such as iron, and elevated temperatures, which occur when the pathogen transitions from mucosal surfaces to deeper tissues or the bloodstream or, when temperatures raise due to host responses (fever) (White-Ziegler et al., 2007; Gant Kanegusuku et al., 2021). To withstand these stress factors and evade the host immune system, *K. pneumoniae* has evolved several adaptive strategies, including the production of a protective capsule and the expression of various virulence factors with distinct functions (Domenico et al., 1994; Cortés et al., 2002b; Paczosa and Mecsas, 2016). In addition to well-characterized virulence factors such as lipopolysaccharides (LPS), iron-chelating complexes like siderophores, and type 1 and type 3 fimbriae, *K. pneumoniae* can synthesize other important molecules including proteins that also contribute to its virulence (Bullen et al., 1972; Gerlach et al., 1989; Miethke and Marahiel, 2007). Examples of outer membrane proteins (OMPs) that play a role in *K. pneumoniae* virulence include the peptidoglycan-associated protein Pal, the murein lipoprotein LppA, and complement factor-binding proteins such as the porin OmpK36 (Albertí et al., 1995; Albertí et al., 1996; Tsai et al., 2011; Hsieh et al., 2013). These murein-interacting proteins are essential for the defense of *K. pneumoniae* against antibiotics and in the maintenance of cell wall integrity (Hsieh et al., 2013). Furthermore, Pal and LppA are involved in protecting the pathogen against killing by human serum components, phagocytosis, and they contribute to the induction of inflammation (Hsieh et al., 2013). While proteomic data on *K. pneumoniae* under zinc (Sukumaran et al., 2021) and iron (Muselius et al., 2020) depletion conditions exist, most knowledge regarding virulence factors and pathogenic mechanisms has been derived from whole genome analyses. These analyses include comparative genomics, genetic screens, molecular analyses of genes and gene clusters, and *in vivo* models, such as murine infection models (Bialek-Davenet et al., 2014; Lam et al., 2021). In contrast, global proteomic data for *K. pneumoniae* remained limited until recently. In fact, an integrative omics study profiled the proteomic patterns of sepsis causing *K. pneumoniae* ST2121 and ST258, providing insights into molecular adaptation mechanisms of clinical isolates exposed to human serum after growing in tissue-model medium, which mimicked the sepsis environment (Mu et al., 2023).

The strain *K. pneumoniae* ATCC BAA-2146 (*Kpn*2146), with the sequence type ST11, was first described in 2010 as the first US isolate producing the carbapenemase New Delhi metallo-β-lactamase-1 (NDM-1) (Yong et al., 2009; Centers for Disease Control and Prevention (CDC), 2010; Hudson et al., 2014). This strain was initially isolated from the urine of a patient treated at medical facilities in India and exhibits resistance to a broad range of β-lactams. In an extensive antimicrobial resistance study, *Kpn*2146 was found to be resistant to all 36 tested antibiotics^2^ (American Type Culture Collection, ATCC, 2022), including twelve cephalosporins, four carbapenems and six penicillins, characterizing it as a multidrug-resistant (MDR) strain. Since its discovery, NDM-1 positive *K. pneumoniae* strains have become widespread globally, posing a significant public health threat (Dortet et al., 2014; Li et al., 2014). All of the above makes *Kpn*2146 an ideal strain for investigating its physiology and analyzing its proteome under various physiological conditions.

In this study, we performed the first global proteome profiling of the isogenic capsule-deficient mutant *K. pneumoniae* ATCC BAA-2146Δ*wza* (*Kpn*2146Δ*wza*) to explore the intracellular proteome and exoproteome under infection-relevant stress conditions.

Quantitative mass spectrometry-based proteome analysis was employed to profile proteins with high sensitivity. Our study provides comprehensive global proteome data for three bacterial growth conditions that mimic physiological and infection-relevant environments. These data will aid in understanding the mechanisms of adaptation and pathogenicity and will contribute to the development of novel and effective vaccine formulations.

## 2 Materials and methods

### 2.1 Bacterial strains and growth conditions

The capsule-deficient *K. pneumoniae* strain *Kpn*2146Δ*wza*, containing a spectinomycin resistance selection marker, was used for proteome analysis under infection-mimicking conditions (Zierke et al., 2025). The mutant strain was chosen because there were no significant differences in growth between the capsule mutant and the wild-type strain and to prevent interference from the capsular polysaccharides of the wild-type strain in the LC and mass spectrometry (MS) analyses.

**FIGURE 1:**
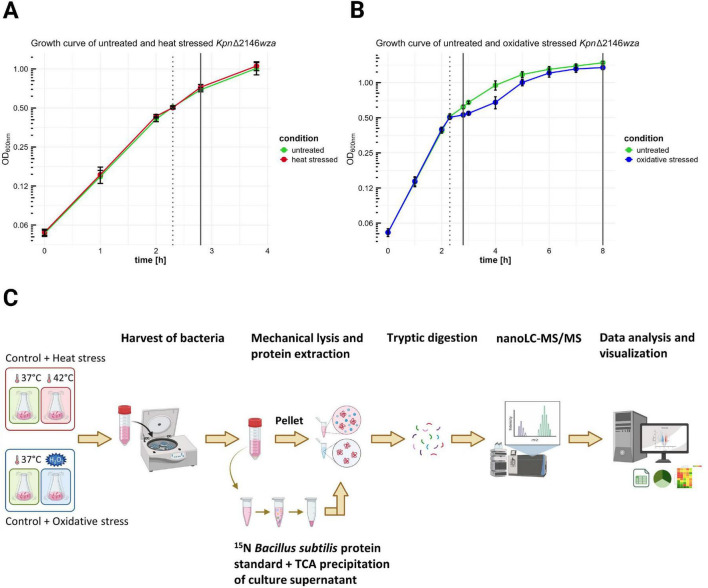
Sampling of bacteria and culture supernatants under untreated, heat shock, and oxidative stress conditions for global proteome analysis. *Kpn*2146Δ*wza* was cultivated in two independent batches under identical growth conditions. The OD of the liquid cultures was measured hourly at 600 nm. Heat shock and oxidative stress were induced by shifting the temperature from 37°C to 42°C and by adding 0.3 mM H_2_O_2_ to the culture, respectively. **(A)** Bacterial harvests from untreated (green) and heat shock (red) cultures were collected 30 min after stress induction for early proteome analysis (dotted line indicates induction time, solid line shows collection time points for both untreated and stressed cultures). **(B)** Sample collections from untreated (green) and oxidative stress (blue) cultures were made 30 min after stress induction and during stationary phase (dotted line indicates induction time, solid lines show collection time points for both untreated and stressed cultures). **(C)** Prior to protein concentration determination and subsequent protease digestion, bacterial cells were lysed mechanically, and proteins were extracted. Supernatant proteins were precipitated with TCA, and a ^15^N labeled *B. subtilis* protein standard was added to the samples. Generated peptides were detected by nanoLC-MS/MS and analyzed using an appropriate database [created in BioRender. “Hammerschmidt, S (2025)” https://BioRender.com/y48f723].

For liquid cultures, the *Kpn*2146Δ*wza* mutant was thawed from a glycerol stock and grown overnight in tryptic soy broth (TSB) containing 100 μg/ml spectinomycin. A 100 μl aliquot of the stock culture was inoculated into 900 μl of TSB, and a 10-step dilution series was prepared, ranging from 10^–1^ to 10^–10^. The five most diluted cultures were then incubated at 37°C and 130 rpm in a shaking incubator (Innova 44^®^, New Brunswick Scientific). A TSB culture from the overnight dilution series that, according to Zierke et al. (2025), was in the exponential phase (OD_600_ ≈ 3), was selected for further cultivations. A volume of this culture enabling inoculation of 20 ml chemically-defined medium (CDM_*i*_) to a starting OD_600_ of 0.1 was transferred into a fresh 1.5 ml reaction tube (Sorenson™ BioScience Inc.) and centrifuged at 5,000 × *g* for 1 min to enable removal of remaining TBS. The resulting bacterial pellet was resuspended in 1 ml of fresh CDM_*i*_ and then added to 19 ml CDM_*i*_ to achieve the 20 ml culture volume for pre-cultivation. This step was necessary to allow the bacteria to adapt and grow in CDM_*i*_ until reaching the late exponential phase after initial growth in rich medium. The pre-culture was incubated at 37°C and 130 rpm in a shaking incubator until it reached late-exponential phase (OD_600_ of approximately 1). The main cultivation for proteome analysis was then inoculated with bacteria from the exponential growth phase, starting with an OD_600_ of 0.05 in fresh CDM_*i*_. The culture was incubated and grown until reaching the mid-exponential and stationary phases (Figures 1A, B). CDM_*i*_ is based on RPMI 1640 medium (Roswell Park Memorial Institute 1,640) with 2.05 mM L-glutamine (Cytiva HyClone™), supplemented with adenine (4 mg/ml), uracil (8 mg/ml) in hydrochloric acid (1 M) and a glucose-containing buffer (74 g/l glucose, 24 g/l sodium hydrogen carbonate, 7.35 g/l sodium dihydrogen phosphate, 1.11 g/l glycine, 0.456 g/l choline chloride) (Schulz et al., 2014). Bacterial growth was monitored hourly until mid-exponential phase (OD_600_ of 0.5), when bacteria were exposed to stresses.

Heat stress was induced by shifting the incubation temperature from 37°C to 42°C, whereas oxidative stress was triggered by the addition of hydrogen peroxide (0.3 mM). The temperature shift to 42°C was selected to simulate human fever conditions and has been employed in previous studies on heat stress (Palau et al., 2023; Müller et al., 2024). The hydrogen peroxide concentration was empirically determined, by testing concentrations of 0.1, 0.3, 0.5, and 1 mM (data not shown). These concentrations were chosen based on previous studies, where varying concentrations were used depending on the experimental design and bacterial strain (Srinivasan et al., 2013; Peng et al., 2024). The temperature shift to 42°C was achieved by transferring the culture to a pre-heated incubator, and heat stress was maintained for 30 min. Sampling after exposure to hydrogen peroxide occurred after 30 min or after approximately 5.7 h, when cells entered the stationary phase. Untreated control cultures were grown for sampling in parallel. All cultures were processed with four independent biological replicates (Figures 1A–C). To ensure a meaningful interpretation of the experimental data, additional growth curves of the capsulated and capsule-deficient strains were recorded under conditions identical to those reported for the mutant strain. Cell counts were assessed at three distinct time points: (1) during mid-exponential phase, prior to stress induction; (2) 30 min after stress induction; and (3) upon entry into the stationary phase (5.7 h after exposure to hydrogen peroxide), to evaluate the long-term effects of oxidative stress on cellular viability. Cell enumeration was conducted using the CASY^®^ TT-2QA-2583 with a 45 μm capillary according to manufacturer’s specifications. Data were obtained from two independent biological replicates, each with two technical replicates, and data analysis was done using CASYworX software version 1.26 (OLS OMNI Life Science GmbH & Co. KG). The growth curves and cell count data are presented in Supplementary Figures 1–3.

### 2.2 Harvesting of bacterial cultures and preparation of samples for intracellular proteome and exoproteome analysis

The *Kpn*2146Δ*wza* strain was harvested 30 min after the induction of heat and oxidative stress during the exponential growth phase. For oxidative stress treated cultures, samples were taken additionally during the entry into stationary phase (5.7 h after addition of hydrogen peroxide). This was done to assess whether *Kpn*2146Δ*wza* could recover or would exhibit persistent changes of its physiological state. Unlike for oxidative stress, the primary focus of the heat stress analysis was on the early adaptive response immediately after stress induction. Untreated cultures were harvested in parallel. In general, 7.5 optical density units (ODUs) were harvested to ensure reproducible cell disruption and availability of similar sample amounts for subsequent processing. A harvest of 7.5 ODUs was selected to standardize the cell quantity, with one ODU corresponding to a specific cell count based on the measured OD (the required harvest volume was calculated by dividing 7.5 by the actual OD). Bacterial cells were centrifuged at 15,000 × *g* for 10 min at 4°C. The supernatant was transferred into a fresh centrifuge tube and shock-frozen in liquid nitrogen to immediately preserve the exoproteome. The bacterial pellet was washed once with phosphate-buffered saline (PBS), then resuspended in 100 μl of denaturing buffer [20 mM HEPES, 2% (w/v) sodium dodecyl sulfate (SDS)]. The suspension was then denatured by shaking at 1,400 rpm and 95°C for 1 min. For intracellular proteome analysis, bacterial cells were mechanically disrupted using a bead mill (Sartorius Stedim Biotech). To achieve this, the resuspended cells were transferred into a Teflon vessel which was cooled in liquid nitrogen and contained a steel ball. The frozen vessel was then shaken at 2,600 rpm for 3 min. The resulting cell powder was resuspended in an equal volume of 20 mM HEPES to reduce the SDS concentration to 1% (w/v), and incubated for 1 min by shaking at 1,400 rpm and 95°C. To remove DNA and RNA, 4 mM magnesium chloride, and 0.005 U/μl benzonase were added and samples were incubated for 5 min in an ultrasonic bath at room temperature. The cell debris was then removed by centrifugation at 17,000 × *g* for 30 min, and the supernatant was transferred into a fresh 1.5 ml reaction tube.

Prior to the digestion of intracellular proteins, the protein concentration was determined using a BCA assay (Micro BCA™ Assay Kit, Thermo Fisher Scientific) in an automated manner with the open science OT-2 liquid handling robot (Opentrons) and the in-house developed analysis pipeline MassSpecPreppy (Reder et al., 2024). A sample volume corresponding to 5 μg of total protein per sample was adjusted with HPLC-grade water (J. T. Baker, Thermo Fisher Scientific) to a final volume of 10 μl for intracellular samples and subjected to a magnetic bead-based single-pot, solid-phase-enhanced sample preparation (SP3) protocol as described previously (Blankenburg et al., 2019; Hughes et al., 2019). Briefly, equal volumes of hydrophobic and hydrophilic beads (GE Healthcare) in HPLC-grade water, with a concentration of 20 μg/μl, were mixed, and 10 μl of this bead mixture was added to each protein sample. A final concentration of 70% (v/v) acetonitrile (ACN) was then added, and the samples were incubated by shaking for 18 min at 1,400 rpm. The samples were placed on a magnetic rack to remove the supernatant. The beads were washed twice with 70% (v/v) ethanol by resuspending the beads and placing the tubes back on the magnetic rack. Two additional wash steps were performed with 100% (v/v) ACN as previously described, before the supernatant was discarded. The air-dried beads with bound proteins were resuspended in 20 mM ammonium bicarbonate buffer containing trypsin (Promega) at an enzyme-to-protein ratio of 1:25 and incubated overnight (16 h) at 37°C for digestion. The digestion was terminated by adding 95% (v/v) ACN and shaking the samples at 1,400 rpm for 15 min. Following the removal of ACN using the magnetic rack, the peptides were eluted from the magnetic beads in 2% (v/v) DMSO (Sigma-Aldrich) in HPLC-grade water and transferred to new 1.5 ml reaction tubes. The tubes were placed back on the magnetic rack before transferring the supernatant to an MS vial, ensuring that no remaining beads were transferred. The peptide solution in the MS vial was then mixed with an equal volume of a buffer containing 4% (v/v) ACN and 0.2% (v/v) acetic acid in HPLC-grade water for subsequent nanoLC-MS/MS analysis.

The exoproteome samples were thawed, and 2 ml of supernatant from each sample were precipitated with 15% (w/v) trichloroacetic acid (TCA) for 48 h at 4°C. After precipitation, the samples were centrifuged at 17,000 × *g* for 1 h and 4°C. The supernatant was discarded, and the remaining pellet was washed twice with 100 μl of pre-cooled 70% (v/v) ethanol for 10 min at 17,000 × *g* and 4°C. The ethanol was removed, and the pellet was air-dried for 1 min. The pellet was then resuspended in 40 μl of 20 mM HEPES with 1% SDS (w/v) and incubated for 5 min by shaking at 1,400 rpm and 65°C. Protein concentration was determined using the BCA assay as previously described. A ^15^N isotope-labeled protein standard was added to 2 ml of freshly thawed supernatant at a concentration equivalent to 15% of the mean protein concentration of all supernatant samples. The samples containing the 15% ^15^N isotope-labeled protein standard were then precipitated, and protein concentration was calculated as described earlier.

To produce the heavily labeled ^15^N standard, *Bacillus subtilis* (*B. subtilis*) BSB1 cells (Nicolas et al., 2012) were cultivated in a 1:10 dilution series up to 10 dilution levels in BioExpress^®^ Bacterial Cell Media (U-^1^5N, 98%) (CGM-1000-N - Cambridge Isotope Laboratories, Inc.) overnight at 37°C and with shaking at 220 rpm in a shaking incubator (Innova 44^®^, New Brunswick Scientific). The following morning, an exponentially growing culture was used to inoculate a main culture in the ^15^N-containing medium to an initial OD_540_ of 0.05. Growth was monitored photometrically, and the entire culture was harvested at the mid-exponential growth phase (OD_540_ = 1.5). The cells were pelleted by centrifugation (3 min at 4°C and 6,900 × *g*) and washed with 20 mM HEPES pH 8.0. The pellets were lysed using a bead mill, followed by SDS denaturation under heat and subsequent benzonase digestion to eliminate nucleic acids. Protein concentration was determined using the BCA assay as described earlier. The protein concentration of the ^15^N standard was 1.42 μg/μl.

A sample volume corresponding to 1.7 μg total protein per precipitated sample was adjusted with HPLC-grade water to a total volume of 40 μl and subjected to the SP3 protein digestion protocol as described previously, using 2.55 μl of the bead mixture. The digestion was stopped with 0.5% (w/v) trifluoroacetic acid (TFA). The samples were then centrifuged for 2 min at 17,000 × *g*, after which they were placed on a magnetic rack. The clear supernatant was transferred into a fresh 1.5 ml reaction tube and centrifuged again for 2 min at 17,000 × *g*. Finally, the samples were placed on a magnetic rack, and 20 μl of the peptide solution was transferred to an MS vial for nanoLC-MS/MS analysis.

### 2.3 NanoLC-MS/MS data acquisition

The peptides were separated by the UltiMate™ 3000 RSLCnano system (Thermo Fisher Scientific), equipped with a trap column (75 μm inner diameter, 3 μm C18 particles; Acclaim PepMap100, Thermo Fisher Scientific) and an analytical column (25 cm × 75 μm, 2.6 μm C18 particles; Accucore, Thermo Fisher Scientific). The elution was performed using a solvent system consisting of solvent A [0.1% (v/v) acetic acid in HPLC-grade water] and solvent B [100% (v/v) ACN in 0.1% (v/v) acetic acid]. After ionization of the separated peptides, MS/MS analysis was conducted on a Q Exactive HF mass spectrometer (Thermo Fisher Scientific) for intracellular samples or an Exploris 480 mass spectrometer (Thermo Fisher Scientific) for supernatant samples, both in data-independent (DIA) mode. The samples were measured randomized to minimize batch effects during the analysis. Additional details on nanoLC-MS/MS acquisition can be found in Supplementary Table A. The MS proteomics data have been deposited in the ProteomeXchange Consortium *via* the PRIDE partner repository (Perez-Riverol et al., 2022) with the dataset identifier PXD052921.

### 2.4 Data analysis

The raw MS data was analyzed using the Spectronaut^®^ software (version 18.2.230802.50606 for intracellular proteome, 18.6.231227.55695 for exoproteome) (Biognosys AG) in directDIA mode, with searches conducted against the NCBI database entry of the annotated *Kpn*2146 genome (NCBI accession CP006659.2, 01/25/2022, 5,148 entries). Methionine oxidation was specified as a variable modification. The analysis selected peptides that were digested with trypsin (Trypsin/P), with a minimum peptide length of seven amino acids and a maximum of 52 amino acids. A precursor q-value cutoff of 0.001 and a maximum of two missed cleavages were applied. Detailed search settings for intracellular proteome data acquisition are provided in Supplementary Table B.

For the supernatant samples containing a ^15^N isotope-labeled protein spike-in standard, a library search using a ^15^N *B. subtilis* library with 21,727 targeted precursors was applied, enabling local normalization to the *B. subtilis* standard only. Additionally, a *K. pneumoniae* light-ion library with 61,348 targeted precursors was created from all samples processed in this project, along with the *Kpn*2146 protein database. The settings for the exoproteome data analysis using spectral libraries are detailed in Supplementary Table C.

For protein quantification, Spectronaut’s iBAQ (intensity Based Absolute Quantification) intensity values were used (Schwanhäusser et al., 2011). Data analysis was performed on proteins detected with at least two peptides in at least one replicate using the SpectroPipeR package using R^3^ (version 4.1.3) and tidyverse (version 1.3.2) in combination with several R packages, as described as follows (Wickham et al., 2019; Michalik et al., 2025). Only *K. pneumoniae* proteins were included in the statistical analysis. To generate peptide intensity data, the sum of ion intensities per sample and peptide was calculated. Statistical analysis at the peptide level was conducted using the ROPECA (Reproducibility-optimized Peptide Change Averaging) approach (PECA R package version 1.30.0) (Elo et al., 2008; Suomi and Elo, 2017). Statistical results are provided in Supplementary Table 1. Significant changes in protein intensities were defined by an absolute fold change ≥ 1.5 and a Benjamini-Hochberg adjusted *p*-value (q-value) ≤ 0.05 (multiple testing correction) (Benjamini and Hochberg, 1995; Yekutieli and Benjamini, 1999). Data visualization was performed using the R packages tidyverse (version 1.3.2) and ggrepel^4^ (version 0.9.2). Following analyses were conducted with R version 4.3.3. For further data visualization, additional R packages devtools^5^ (version 2.4.5), moonbook (version 0.3.4), webr^6^ (version 0.1.6), doBy^7^ (version 4.6.21), and paletteer^8^ (version 1.3.9) (Moon, 2015; Wickham, 2016) were used.

The subcellular localization of *Kpn*2146 proteins was predicted using the DeepLocPro subcellular localization prediction tool (version 1.0) (Moreno et al., 2024). The amino acid sequences of the annotated proteins, obtained from the NCBI database, were submitted to DeepLocPro, which predicted the subcellular localization for each protein (prediction probability threshold set to ≥ 0.7). For functional assignment analysis, the TheSEED database for *Kpn*2146 was retrieved in November 2022 (Overbeek et al., 2014). To associate functional annotations with the protein identifiers, Roary (version 3.13.0) was used to identify orthologous annotations between the RefSeq annotation (GCF_000349285) and GenBank annotation (GCA_000349285) (Page et al., 2015).

## 3 Results and discussion

### 3.1 Impact of stress on growth behavior and overview of the global proteome analysis

A global proteome analysis of *K. pneumoniae* can significantly contribute to the understanding of its pathogenic mechanisms. Preferably, we would have liked to analyze the *Kpn*2146 wild-type strain, but encountered difficulties during the analysis by mass spectrometry likely due to contaminants originating from the capsule. Therefore, we assessed the impact of stresses on the wildtype *Kpn*2146 and its isogenic Δ*wza* mutant. These experiments established that the *Kpn*2146 wild-type and the capsule-mutant *Kpn*2146Δ*wza* strain exhibited no significant differences in growth behavior and survival during stress exposure (growth curves are provided in Supplementary Figure 1). Thus, we decided to perform the study with the *Kpn*2146Δ*wza* strain. To simulate the impact of nutrient restriction, we analyzed the intracellular proteome and exoproteome of *Kpn*2146Δ*wza* under infection-mimicking stress conditions using a chemically-defined medium (CDM_i_) (Schulz et al., 2014). The bacterial growth was unaffected by the heat shock employed (Figure 1A), but oxidative stress caused a temporary growth reduction, followed by recovery (Figure 1B).

**FIGURE 2:**
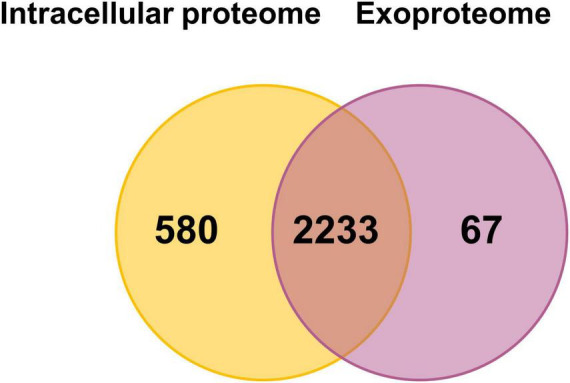
Number of proteins detected with at least two peptides in the intracellular proteome and exoproteome of *Kpn*2146Δ*wza*. Venn diagram showing the number of proteins detected under all conditions in the intracellular proteome (orange) and the exoproteome (violet).

We detected over 2,800 proteins in the bacterial cell pellet and 2,300 proteins in the exoproteome, covering 54 and 45% of the annotated genome, respectively. A total of 2,233 proteins were found in both compartments, whereas 580 and 67 were unique to the intracellular proteome or exoproteome, respectively (Figure 2). Among the 580 proteins unique to the intracellular proteome, multidrug resistance proteins (MdtD, Stp_3) and the LPS biosynthesis protein Wzx were identified. The 67 proteins seen in the exoproteome include fimbrial proteins (FimA_2, FimA_3), lipoproteins (OsmB, MetQ), and ABC transporters (OppA_1, YtfQ_1, YtfQ_2).

Detecting such a large number of proteins in the exoproteome was unexpected and surprising. We assume that a large part of the proteins detect in the exoproteome originates from lysis of a limited fraction of cells both during growth and during stress exposure. These proteins were also accessible due to the excellent sensitivity of the MS. The much lower mean iBAQ intensities of cytoplasmic proteins in the exoproteome compared to those in the intracellular proteome indicate that indeed limited cell lysis is always observed. A recent study identified 1,177 proteins in *K. pneumoniae* outer membrane vesicles (OMVs) using MS, which may contribute to the high number of proteins detected in our analysis (Hua et al., 2022). Furthermore, the accumulated intensities of all 1,703 proteins of the exoproteome originating from the cytosol make up only 37% of the total protein intensities of the exoproteome, whereas roughly 335 truly secreted proteins make up 50% of the total signal intensity of the exoproteome. To determine the impact of cell lysis under the growth conditions tested independent of the proteome analysis, we performed counting of viable bacterial cells to validate our data. The cell counts for every analyzed condition are provided in Supplementary Figure 3. The recorded values ranged from 4.2 × 10^8^ to 5.09 × 10^9^ cells/ml. We observed an increase in cell numbers per ml as the culture progressed from the exponential to the stationary phase, which is consistent with the observed bacterial growth pattern. However, upon stress induction, there was no significant decrease in cell numbers per ml, except for the lower numbers after exposure to oxidative stress originating from the reduced growth rate. Thus, stresses did not lead to increased cell lysis or cell death.

Ranked iBAQ values were used to compare protein abundances. A comprehensible table highlighting the top ten most abundant proteins for facilitated interpretation and comparison across conditions with their respective functions and pathways is provided in Supplementary Table D. The iBAQ intensities of all proteins per condition and compartment are provided in Supplementary Table 2. One of the most abundant proteins across conditions was the major outer membrane (OM) lipoprotein Lpp in both, the intracellular proteome and exoproteome. Lpp is a conserved virulence factor of *K. pneumoniae* that is important for serum resistance and protection from phagocytosis (Hsieh et al., 2013). Ribosomal proteins, along with the OMPs OmpA and OmpX, were also highly abundant, contributing to bacterial adherence, invasion of host cells, and resistance to host immune defenses (Prasadarao et al., 2002; Sukumaran et al., 2003). In the intracellular proteome, the DNA-binding protein HU-alpha HupA also showed high abundances. HupA forms one of the two subunits of the histone-like protein HU and is essential for colonization of the urinary tract, the primary site of *Kpn*2146 tissue infection (Wada et al., 1988; Struve et al., 2003; Hudson et al., 2014).

Unlike the intracellular proteome, the metal-binding protein ZinT showed high abundance in the exoproteome under both untreated and stress conditions. ZinT is involved in zinc acquisition, crucial for pathogen growth during infection (Petrarca et al., 2010; Maunders et al., 2024). For *E. coli* it has been shown that ZinT is strongly induced upon adhesion to epithelial cells and can be secreted to sequester zinc from the host environment (Gabbianelli et al., 2011), which could explain why ZinT was found highly abundant in the exoproteome but not in the intracellular proteome (rank 81-175).

### 3.2 Proteome adaptation of *K. pneumoniae* under physiological infection-relevant stress conditions

The analysis of altered protein abundances under infection-relevant physiological stress conditions will contribute to an improved understanding of the adaptive stress responses of pathogens like *K. pneumoniae* to the host environment during infection.

**FIGURE 3:**
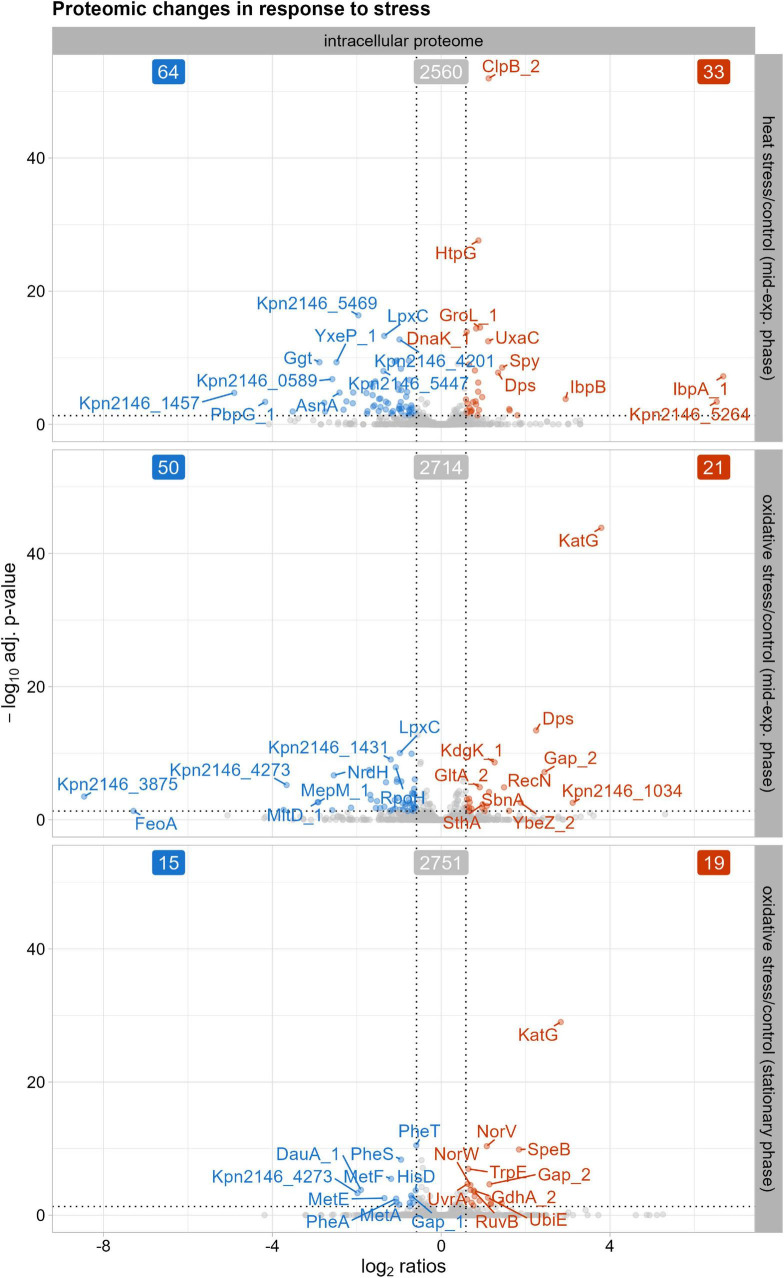
Proteins with significantly altered abundances in the intracellular proteome after stress exposure compared to untreated growth conditions. The top ten proteins, determined by q-value, with either decreased (blue) or increased (red) abundances are labeled. The cutoff for significant changes in abundance was set to an absolute fold change of ≥ 1.5 and a q-value of ≤ 0.05. Significantly altered protein abundances 30 min after heat shock, 30 min after oxidative stress, and after oxidative stress in stationary phase.

Our intracellular proteome data after heat shock induction revealed an early stress response (Figure 3). Thirty-three proteins showed a significant increase in abundance 30 min after stress induction, including chaperones such as ClpB_2, DnaK_1, GroL_1, and HtpG. Small heat shock proteins IbpA_1 and IbpB also showed increased abundance, marking the early physiological reaction to heat shock. Other general stress proteins, like DNA protection protein Dps and spheroplast protein Y precursor Spy, also showed increased abundances after heat shock. In *E. coli*, Dps plays a crucial role in protecting against external stresses such as heat stress (Park et al., 2023). Dps has been identified for its ferroxidase and DNA-binding activity, as well as its role in regulating stress resistance gene expression during heat shock (Almirón et al., 1992; Ilari et al., 2002; Park et al., 2023). Spy, identified as a periplasmic chaperone in *E. coli* (Quan et al., 2011), assists in protein folding to prevent protein aggregation under cell envelope stress, which could indicate induced envelope stress in *K. pneumoniae* after heat stress due to disrupted OMPs and periplasmic proteins. Sixty-four proteins showed a significant decrease, including the extended spectrum beta-lactamase Kpn2146_5469 and components of a multidrug efflux system like Kpn2146_4201.

Thirty minutes after oxidative stress induction, 50 proteins decreased in their abundance, while 21 proteins showed an increase in the intracellular proteome (Figure 3). Among the proteins displaying decreased levels compared to the untreated condition were those involved in cell wall biosynthesis and peptidoglycan remodeling, such as the membrane-bound lytic murein transglycosylase D precursor MltD_1 and the murein DD-endopeptidase MepM_1. Similarly, a putative periplasmic iron-binding protein precursor Kpn2146_4273 and the ferrous iron transport protein FeoA showed decreased levels, indicating an adaptation of cell wall biosynthesis and iron metabolism regulation during oxidative stress. The 21 proteins with increased abundance after oxidative stress induction included the general stress protein Dps and the DNA repair protein RecN. Dps resembles ferritin and can scavenge iron, thus preventing damage by the Fenton reaction to DNA (Fenton, 1876; Ilari et al., 2002). As part of the oxidative stress response, catalase-peroxidase KatG increased in abundance compared to untreated conditions. In addition to specific and unspecific stress proteins, the putative siderophore biosynthesis protein SbnA also showed increased levels. SbnA, identified in the *S. aureus sbn* cluster, is involved in the synthesis of staphyloferrin B, a siderophore synthesized under iron deprivation (Dale et al., 2004; Kobylarz et al., 2016). To limit reactive oxygen species formed through the Fenton reaction, siderophore-mediated iron acquisition is inhibited (Escolar et al., 1999). However, Nobre and Saraiva (2014) showed that staphyloferrin B increases resistance to oxidative stress *via* its transport system. This suggests that a similar siderophore system in *K. pneumoniae* could help provide resistance against oxidative stress.

In the stationary phase after oxidative stress induction, 15 proteins showed a decrease in abundance compared to untreated conditions (Figure 3). These included proteins involved in amino acid metabolism, such as phenylalanine biosynthesis proteins (PheA, PheS, PheT) and methionine biosynthesis proteins (MetA, MetE, MetF). *K. pneumoniae* must adjust metabolic processes to conserve energy and enhance survival in nutrient-limited stationary phase. Since no data are available regarding the reduction of phenylalanine and methionine levels in oxidative stressed Gram-negative bacteria, our data suggests that *K. pneumoniae* downregulates methionine and phenylalanine synthesis, possibly due to prioritize other amino acid pathways for pathogen survival during stationary phase. In contrast, 19 proteins showed an increase in abundance after oxidative stress, including KatG, as observed in the mid-exponential phase. Other proteins with increased levels included those involved in DNA repair systems, such as the UvrABC system protein A UvrA and the Holliday junction ATP-dependent DNA helicase RuvB, indicating activation of DNA repair mechanisms after the initial oxidative stress response.

**FIGURE 4:**
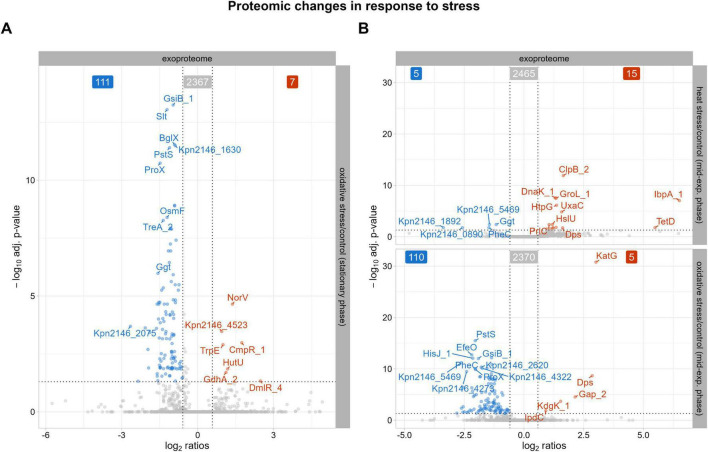
Proteins with significantly altered abundances in the exoproteome after stress exposure compared to untreated growth conditions. The top 10 proteins, determined by q-value, with either decreased (blue) or increased (red) abundances are labeled. The cutoff for significant changes in abundance was set to an absolute fold change of ≥ 1.5 and a q-value of ≤ 0.05. **(A)** Significantly altered protein abundances after oxidative stress during stationary phase. **(B)** Significantly altered protein abundances 30 min after heat shock and 30 min after oxidative stress.

Our exoproteome data, shown in Figure 4A, revealed decreased abundances of proteins associated with cellular osmoregulation in the stationary phase after oxidative stress. These include the putative osmoprotectant uptake system substrate-binding protein OsmF, periplasmic trehalase TreA_2, and the glycine betaine-binding periplasmic protein ProX (Boos et al., 1987; Dattananda and Gowrishankar, 1989). As the stationary phase exhibited the most prominent proteomic changes, the subsequent discussion will focus exclusively on these findings. In contrast, alterations observed after heat stress were less pronounced (as shown in Figure 4B) and overlapped with changes already identified in the intracellular proteome, while oxidative stress resulted in a broad decrease in protein abundances similar to that observed during the stationary phase. Therefore, these results will not be discussed further. As bacteria prioritize essential functions in the stationary phase, osmoregulation may be temporarily downregulated to allocate resources for maintaining cellular integrity following oxidative stress. The stress response protein gamma-glutamyl transpeptidase Ggt also showed decreased abundances, indicating stress adaptation and recovery, which was supported by the growth curve data. Unlike the intracellular proteome, the fimbrial protein Kpn2146_4523 showed increased abundance in the stationary phase in the exoproteome. Protein-protein BLAST alignment of Kpn2146_4523 revealed 100% identity to the type 3 fimbria major subunit MrkA from *Gamma-proteobacteria* (including *K. pneumoniae*) which is documented in Supplementary Table F (Altschul et al., 1997). MrkA facilitates biofilm formation, a crucial process for *K. pneumoniae* pathogenesis in respiratory and urinary tract infections (Langstraat et al., 2001). The increased abundance of Kpn2146_4523 may reflect the bacteria’s enhanced protective measures against oxidative stress.

**FIGURE 5:**
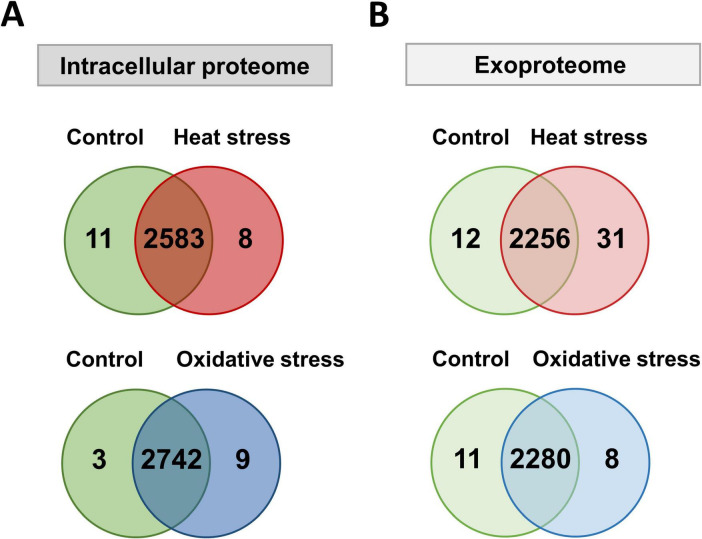
Number of proteins detected with at least two peptides in the intracellular proteome and exoproteome of *Kpn*2146Δ*wza* after stress induction. **(A)** Venn diagrams depicting the number of proteins detected in the intracellular proteome under untreated (green), heat shock (red), and oxidative stress (blue) conditions. **(B)** Venn diagrams depicting the number of proteins detected in the exoproteome under untreated (green), heat shock (red), and oxidative stress (blue) conditions.

To further explore the differences in our comparative analysis, we examined the intracellular proteome and exoproteome separately. Analysis of the intracellular proteome showed eight proteins uniquely detected after heat shock, including small heat shock protein IbpB, multiple antibiotic resistance protein MarA, FabG_8, Kpn2146_0808, Kpn2146_2741, Kpn2146_4746, Kpn2146_5226, and Kpn2146_5264 (Figure 5A). Besides its chaperone activity, IbpB plays a role in adherence and biofilm formation and thus in the pathogenesis of *K. pneumoniae* (Allen et al., 1992; Laskowska et al., 1996; Kuczyńska-Wiśnik et al., 2010; Bao et al., 2022). Nine unique proteins were detected after oxidative stress: PspF_1, FadE, RipA_1, Kpn2146_2870, Kpn2146_3535, HisM_2, DmlR_25, Cbs, DgoA_2.

Interestingly, IbpB was also identified in the exoproteome post-heat shock, confirming a heat stress dependent regulation (Kuczyńska-Wisńik et al., 2001; Figure 5B). Additionally, small heat shock proteins IbpA_1 and IbpA_2 were found in the exoproteome, highlighting the presence of virulence-associated proteins during heat stress. Proteins involved in stress adaptation, such as YieH and AhpD, were identified after oxidative stress, indicating a complex response to environmental challenges, along with others (Kpn2146_2225, PcaR_2, Pcm, IldR_1, SinR_3, and Kpn2146_5382). This first analysis shows that virulence-associated proteins predominantly appeared after heat stress, especially in the exoproteome.

### 3.3 High abundant outer membrane proteins of *K. pneumoniae* are associated with virulence

**FIGURE 6:**
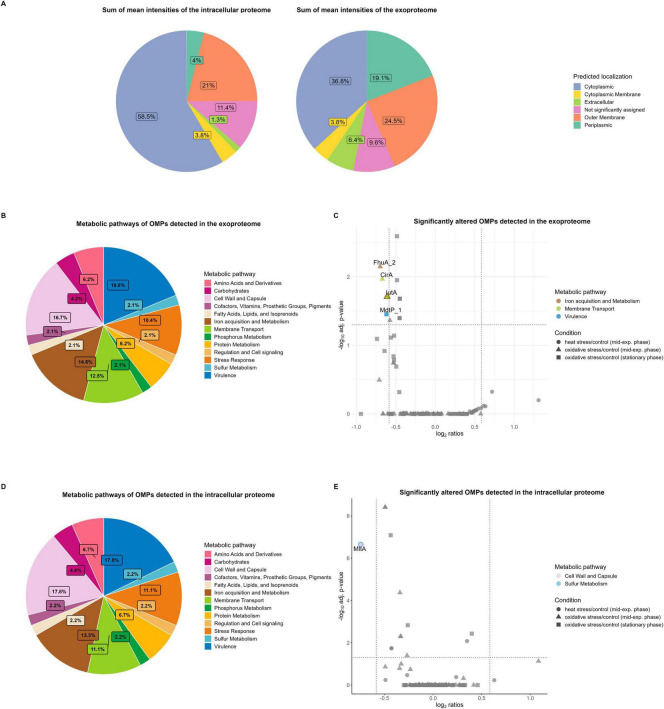
Subcellular localization and metabolic pathways of the total intracellular proteome and exoproteome of *Kpn*2146Δ*wza*. **(A)** Subcellular localization of detected proteins in the intracellular proteome and exoproteome based on the sum of mean protein intensities. **(B)** Metabolic pathway distributions of OMPs detected in the exoproteome. **(C)** Significantly altered metabolic OMPs in the exoproteome under stress conditions (cutoffs: absolute fold change ≥ 1.5, q-value ≤ 0.05). The bicolored protein IutA is functionally annotated to both membrane transport and iron acquisition metabolism. **(D)** Metabolic pathway distributions of OMPs detected in the intracellular proteome. **(E)** Significantly altered metabolic OMPs in the intracellular proteome under stress conditions (cutoffs: absolute fold change ≥ 1.5, q-value ≤ 0.05). The bicolored protein MltA is functionally annotated to both sulfur metabolism, as well as cell wall and capsule metabolism.

Analysis of the subcellular localization of detected proteins in the cellular proteome and exoproteome was performed using the DeepLocPro prediction tool (version 1.0, results downloaded 03/13/2025) (Moreno et al., 2024). Our results revealed increased intensities of extracellular, OM, and periplasmic proteins in the exoproteome, which accounted for one half (50%) of the mean protein intensities in the supernatant of *K. pneumoniae* (Figure 6A). While some cytoplasmic proteins also showed high intensities, the fraction of total intensity assigned to cytoplasmic proteins (36.8% of total intensity, 1,703 cytoplasmic proteins) was much lower compared to that observed in the cellular proteome (58.5% of total intensity, 1,996 cytoplasmic proteins), as expected. Lpp as highly abundant OMP represents 22% of the mean intensities of assigned OMPs in the intracellular proteome and 26% in the exoproteome, supporting the observation of increased OMP intensities in the exoproteome.

Approximately 39% of the assigned OMPs in the intracellular proteome and 38% of those in the exoproteome are involved in at least one metabolic pathway of *K. pneumoniae*. This metabolic functional annotation was derived from the TheSEED database. Pathway analysis of the OMPs showed that most proteins are involved in virulence, cell wall and capsule metabolism, as well as iron acquisition and metabolism (Figures 6B, D). A table with additional information on the number of all proteins involved in metabolism is provided in Supplementary Table E.

In the intracellular proteome, virulence-associated proteins, such as the membrane-bound lysozyme inhibitor of C-type lysozyme and the multidrug-resistant OMP MdtP_1 showed high abundances. Additionally, receptors involved in ferric enterobactin uptake, such as FepA_2, as well as the aerobactin siderophore receptor IutA, were highly abundant within the iron metabolism pathway. Other highly abundant OMPs associated with cell wall and capsule metabolism include the OM beta-barrel assembly machinery (BAM) proteins BamABCDE, which together form the BAM complex. BamBCDE are lipoproteins involved in the insertion of integral beta-barrel proteins into the OM and essential for the survival of *K. pneumoniae* (Sandoval et al., 2011; Svanberg Frisinger et al., 2021).

In the exoproteome, proteins of the BAM complex, the catecholate siderophore receptor Fiu, IutA, and ferrichrome-iron receptor FhuA_3 exhibited high abundances. While these proteins are not classified under the virulence pathway of the TheSEED database, they possess virulence-associated functions during host infection. The high abundant siderophore receptors IutA and FhuA are important virulence factors involved in iron uptake, which is restricted by the host, due to competition for iron with the host (Holden et al., 2018; Muselius et al., 2020). Iron limitation during infection is a key host strategy to limit bacterial growth, as iron is essential for several cellular processes, including DNA replication and transcription, as well as serving as a cofactor for enzymes contributing to the oxidative stress response (Andrews et al., 2003; Muselius et al., 2020). Thus, highly specific mechanisms for iron uptake are critical for *K. pneumoniae* pathogenesis.

Our analysis of significantly altered metabolic OMPs revealed that no OMPs assigned to virulence showed elevated levels during physiological stress conditions compared to untreated conditions (Figures 6C, E). In the intracellular proteome, the membrane-bound lytic transglycosylase A MltA, a lipoprotein involved in sulfur metabolism and cell wall and capsule metabolism, decreased in abundance 30 min after heat shock (Figure 6E). MltA, as a murein hydrolase, is essential for reorganization of the cell wall structure and cross-linked peptidoglycan polymers, which is necessary for bacterial growth (Lommatzsch et al., 1997).

In the exoproteome, OMPs associated with virulence constitute one of the largest proportions among the classified metabolic pathways (Figure 6B). This may, in part, be attributed to passive leakage and the production of OMVs. OMVs are formed through shedding of the OM and consist of components such as periplasmic lipids and proteins (Hussein et al., 2023). These vesicles serve as a transport mechanism for various molecules, including virulence factors, genetic material, and proteins from the cytoplasm and inner membrane (Hussein et al., 2023). A recent study identified 1,177 proteins in *K. pneumoniae* OMVs *via* MS analysis, which may also explain the high number of detected global proteins, in addition to virulence gene cargo (Hua et al., 2022). Regarding iron metabolism, both FhuA_2 and IutA decreased in abundance 30 min after oxidative stress induction, compared to untreated conditions (Figure 6C). The decrease of these receptors, leading to reduced iron uptake, can be explained by the addition of hydrogen peroxide to the *Kpn*2146Δ*wza* culture in exponential growth phase. During entry into stationary phase, no siderophore receptors exhibited decreased abundances. Similarly, the colicin I receptor CirA was reduced under oxidative stress in exponential growth phase. CirA, an OMP with a transmembrane domain, transports iron-bound siderophores (catecholates) into the cell and likely decreased due to hydrogen peroxide exposure (Buchanan et al., 2007; Lan et al., 2022). In the stationary phase, putative multidrug resistance OMP MdtP_1 showed reduced protein levels under oxidative stress conditions compared to untreated conditions, indicating a reduced virulence upon entering the stationary phase due to nutrient limitations and an energy-efficient metabolism of essential cellular processes.

In conclusion, the most abundant OMPs are those involved in pathogenicity-related pathways such as iron acquisition and virulence. The second most prominent pathway, the cell wall and capsule metabolism, also includes proteins essential for *K. pneumoniae* survival, such as those involved in LPS assembly and OMP formation. The fact that these proteins are not significantly altered after stress induction suggests that the high levels persist under various physiological stress conditions, including those encountered during host infection. This finding is important for future drug development and the discovery of vaccine candidates. Potential vaccine candidates should not only be surface-exposed for rapid detection by the host immune system but also be highly abundant, particularly if they are pathogen-specific. To trigger the host’s immune response, vaccine candidates must be immunogenic. Although the capsule may obscure OMPs, recent studies have shown that their immunogenic potential remains significant. For instance, major OMPs such as OmpA, OmpK17, and OmpK36, have been shown to confer protection against *K. pneumoniae* in murine models (Babu et al., 2017; Hussein et al., 2018). Similarly, an OM lipoprotein from encapsulated *Neisseria meningitidis* demonstrated immunogenic properties (Fletcher et al., 2004). Furthermore, a study reported that immunization of mice with a purified protein from a non-encapsulated *K. pneumoniae* strain provided protection against infection by an encapsulated strain (Serushago et al., 1989). These findings suggest that the capsule does not necessarily prevent the recognition of extracellular proteins, as capsule expression is dynamic and influenced by factors such as capsule shedding or reduced expression upon epithelial adherence (Cortés et al., 2002a). Nevertheless, future studies are needed to identify and validate immunogenic proteins from our analysis.

### 3.4 High abundant outer membrane lipoproteins

**FIGURE 7:**
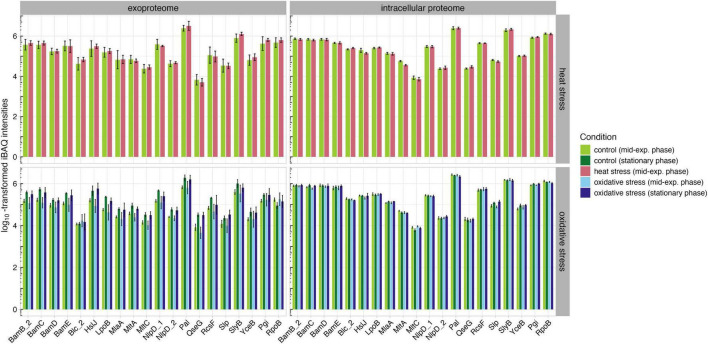
Abundances of OM lipoproteins and housekeeping proteins Pgi and RpoB in the intracellular proteome and exoproteome under untreated (green), heat stress (red), and oxidative stress (blue) conditions.

An *in silico* analysis of conserved OM lipoproteins revealed several lipoproteins that merit detailed analysis to assess their immunogenicity and potential as vaccine candidates (data not shown). Based on this analysis, eighteen lipoproteins were classified as OMPs using DeepLocPro. We then analyzed the abundances of these lipoproteins in the exoproteome and intracellular proteome under physiological stress conditions (Figure 7). To establish baseline protein abundance levels, we selected housekeeping gene products from *K. pneumoniae*, namely Pgi and RpoB, and included these proteins in our analysis (Diancourt et al., 2005). In the intracellular proteome, peptidoglycan-associated lipoproteins Pal and SlyB showed the highest protein levels across all tested conditions. Notably, no significant differences were observed between the two growth phases in the intracellular proteome. In contrast, the exoproteome showed more variation suggesting that the observed changes are more likely responses to nutrient limitations that occur in stationary phase and affect the OMPs. However, Pal and SlyB also exhibited the highest intensity levels across all conditions.

The high abundance of these lipoproteins under all conditions could be valuable for future immunoproteomic studies, as a conserved lipoprotein that remains consistently abundant across various physiological conditions may be of significant interest in the development of therapeutic strategies. Pal, as peptidoglycan-associated protein, is crucial for maintaining cell wall integrity and bacterial fitness (Cascales et al., 2002). In a previous study, a *pal*-deficient *K. pneumoniae* strain was shown to have reduced protection from phagocytosis and serum killing, suggesting that Pal could be a potential vaccine candidate or target for anti-infectives (Hsieh et al., 2013). However, it is important to note that Pal shares high homology with Pal in *E. coli;* therefore, further studies are needed to investigate the presence and relevance of Pal in non-pathogenic *E. coli* to avoid potential cross reactivity (Hirakawa et al., 2022). Similarly, SlyB, a peptidoglycan-associated lipoprotein, plays an important role in resistance to antimicrobial drugs (Plesa et al., 2006). A study involving antibodies against peptidoglycan-associated lipoproteins in *Haemophilus* showed a bactericidal effect against the pathogen, suggesting that Pal and SlyB could also represent potential vaccine candidates (Plesa et al., 2006, 108). While passive immunization of mice with Pal and Lpp did not prevent sepsis in a study by Valentine et al. (2006), Hsieh et al. (2013) demonstrated that *K. pneumoniae* strains lacking Lpp and Pal are attenuated but still produced capsular polysaccharides and LPS (Valentine et al., 2006; Hsieh et al., 2013). Therefore, future studies should focus on immunoproteomic analyses of Pal and, especially, SlyB to further investigate their immunogenicity and role in pathogenesis.

## 4 Limitations of the study

A potential limitation of our study is the use of a capsule-deficient strain, which, although allowing for higher resolution of protein detection, may not fully represent the native conditions of encapsulated *K. pneumoniae*. However, we confirmed that the absence of the capsule did not significantly affect bacterial growth or cell counts after stress exposure, suggesting that the overall stress response remains comparable. Another consideration is the secretion of OMVs, which could contribute to the presence of cytoplasmic proteins in the exoproteome. The large number of detected proteins in the exoproteome should be interpreted with caution. Besides, the abundance of the proteins has to be considered in the interpretation of the data and thus, protein intensity ranking and changes in this ranking after stress exposure might enable focusing on functionally important players. Despite these limitations, our study provides valuable insights into the intracellular proteome and exoproteome under stress conditions, enabling further investigations into virulence-associated factors and bacterial adaptation mechanisms.

## 5 Conclusion

The misuse and overuse of antibiotics have contributed to the emergence of multi-resistant and hypervirulent *K. pneumoniae* strains, particularly in vulnerable populations such as newborns and the immunocompromised. In response to these challenges, there is an urgent need for new preventive strategies, including vaccine development. Our study focused on global proteome profiling of *K. pneumoniae* to better understand its pathophysiology under various physiological conditions. The data obtained through powerful MS approaches have the potential to support the development of treatments and vaccines against multidrug-resistant strains such as *K. pneumoniae*.

The detection of over 2,800 proteins across the intracellular proteome and exoproteome not only demonstrates the capacity for protein identification but also highlights proteins with essential roles in *K. pneumoniae* pathophysiology and adaptation to environmental changes. The discovery of proteins like IbpB, exclusively detected after heat shock, underscores their importance in the bacterium’s response to environmental stress and their direct role in enhancing pathogenicity, such as through biofilm formation (Kuczyńska-Wiśnik et al., 2010; Bao et al., 2022). Similarly, proteins like PspF_1, present under oxidative stress conditions, suggest targeted bacterial responses essential for survival and adaptation in hostile host environments (Jovanovic et al., 1996). Furthermore, the high abundance of proteins like Lpp, OmpA, and HupA, which are involved in critical pathogenic processes such as serum resistance and host colonization, points to their pivotal roles in the bacterium’s overall pathogenic profile (Hsieh et al., 2013). The consistent abundance of these proteins under both untreated and stress conditions elucidates the virulence of *Kpn*2146 and identifies potential targets for therapy or disease prevention.

Significant increases in level of chaperones and heat shock proteins shortly after heat stress emphasize their crucial roles in immediate cellular protection and protein homeostasis. Elevated levels of proteins such as Spy and Dps not only facilitate proper protein folding and protection against oxidative damage but also suggest modifications in cell envelope stress responses and iron metabolism under stress conditions (Almirón et al., 1992; Ilari et al., 2002; Quan et al., 2011; Park et al., 2023). The decreased abundances of proteins related to cell wall biosynthesis and iron transport during oxidative stress indicate an adaptive shift that prioritizes stress resistance over growth. Additionally, the oxidative stress response in the stationary phase highlights a strategic downregulation of amino acid synthesis, particularly of phenylalanine and methionine, as an energy conservation mechanism during nutrient starvation. The observed proteome alterations, including increased levels of DNA repair and biofilm-related proteins, demonstrate that *K. pneumoniae* enhances its defense and repair mechanisms, preparing for prolonged stress conditions or contributing to infection persistence.

Our proteome analysis reveals significant adaptations in protein abundances, particularly of OMPs and their involvement in key metabolic pathways crucial for pathogenesis. The majority of high-abundant OMPs in the intracellular proteome and exoproteome are involved in iron acquisition, virulence, and the metabolism of the cell wall and capsule – systems essential for bacterial survival and pathogenicity. Specifically, the consistent high levels of OM lipoproteins Pal and SlyB in both the exoproteome and intracellular proteome under various physiological stress conditions suggest their pivotal roles in bacterial fitness and survival mechanisms such as maintaining cell wall integrity and resistance to antimicrobial agents (Plesa et al., 2006; Hsieh et al., 2013). Moving forward, immunoproteomic analyses focusing on these proteins will be essential to elucidate their immunogenic properties and effectiveness in inducing protective immune responses, thereby advancing the development of vaccines against *K. pneumoniae*.

Overall, our study not only enhances our understanding of the proteomic landscape of *Kpn*2146Δ*wza* but also emphasizes the value of proteomic analyses in unraveling the complexities of *K. pneumoniae* pathogenesis and adaptation.

## Data Availability

The mass spectrometry proteomics data have been deposited to the ProteomeXchange Consortium *via* the PRIDE partner repository with the dataset identifier PXD052921.

## Footnotes

1 https://apps.who.int/iris/handle/10665/112642 (retrieved on 01/10/2022)

2 https://www.atcc.org/-/media/resources/brochures/multidrug-resistant-and-antimicrobial-testing-reference-materials.pdf?rev=a1da5dcf40bf41c883d389c2815fdc66 (retrieved on 01/09/2023)

3
https://www.R-project.org/

4
https://ggrepel.slowkow.com/

5
https://devtools.r-lib.org/

6
https://cardiomoon.github.io/webr/

7
https://github.com/hojsgaard/doby/

8
https://github.com/EmilHvitfeldt/paletteer/
