# Supplementary material for: Global quantitative proteome analysis of a multi-resistant Klebsiella pneumoniae strain
Source: Front Microbiol. 2025 May 19;16:1528869. doi: 10.3389/fmicb.2025.1528869 (PMC12127431; doi:10.3389/fmicb.2025.1528869)
Supplement: Supplementary file 3 [file Data_Sheet_1.pdf]

Supplementary Material

Supplementary Figures

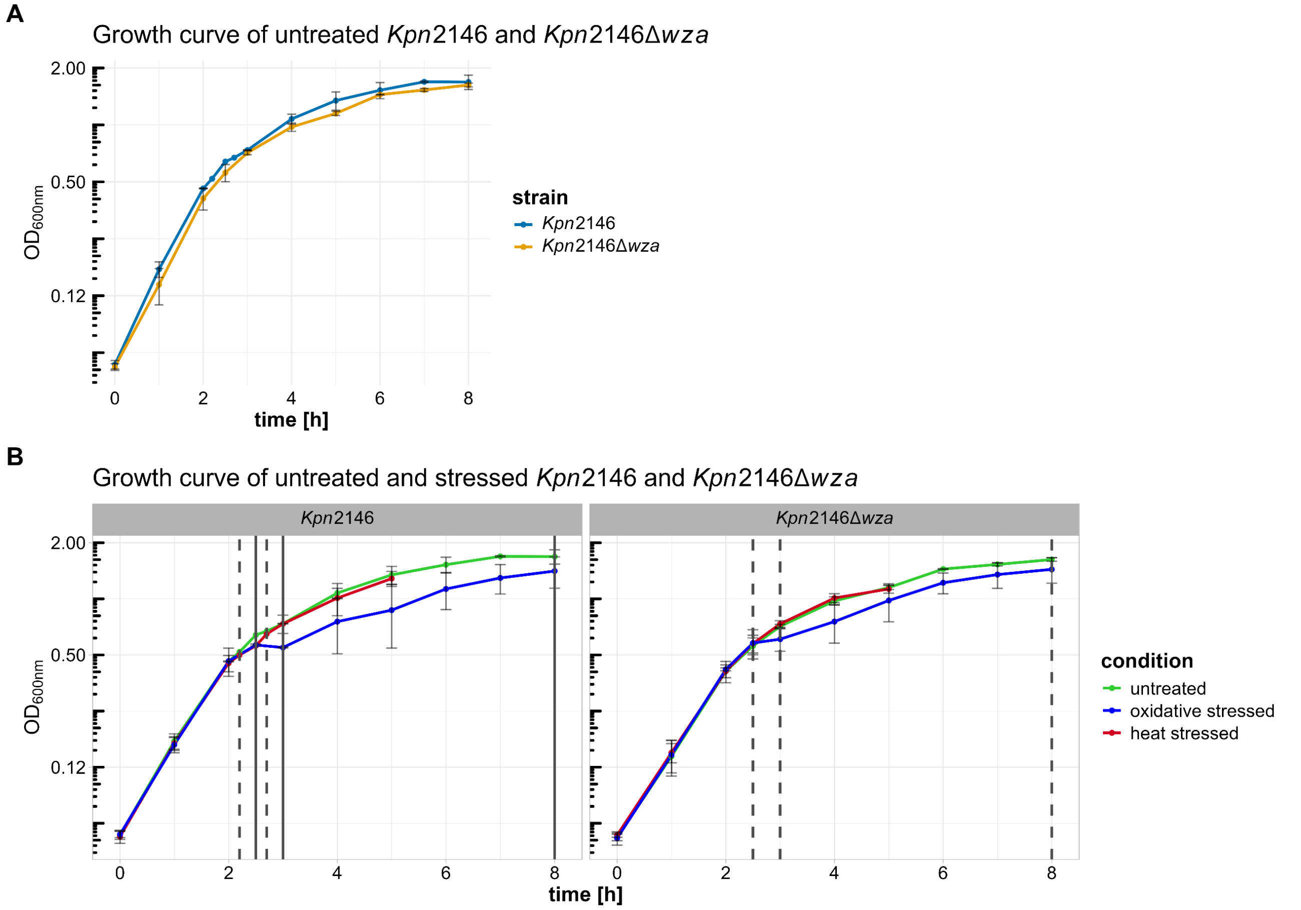

Figure S1 | Growth curves of the encapsulated wild-type *Kpn*2146 and capsule-deficient *Kpn*2146Δ*wza* strains. Panel (A) shows the comparison of growth between untreated *Kpn*2146 and the capsule-deficient mutant *Kpn*2146Δ*wza*. A paired t-test was performed on the growth curve data at each time point, and the two strains exhibited no significant difference in growth. In panel (B), the dashed lines indicate the time points at which the samples were collected for cell counting. The solid lines in the growth plot of *Kpn*2146 represent the induction of stress and subsequent sampling of oxidative-stressed and untreated cultures, due to differences in growth rates. Stress was induced when the cultures reached an OD_{600} of 0.5. Samples were collected at three time points: before stress

induction, 30 min after induction, and at the stationary phase for both untreated and oxidative stressed cultures. A paired t-test was performed on the growth curve data at each time point, and no significant differences in growth were observed between the two strains under any condition. However, when comparing untreated and stress conditions within each strain, significant differences in growth were detected for both the wild-type ($p \approx 0.001$) and the mutant strain ($p \approx 0.005$) following oxidative stress.

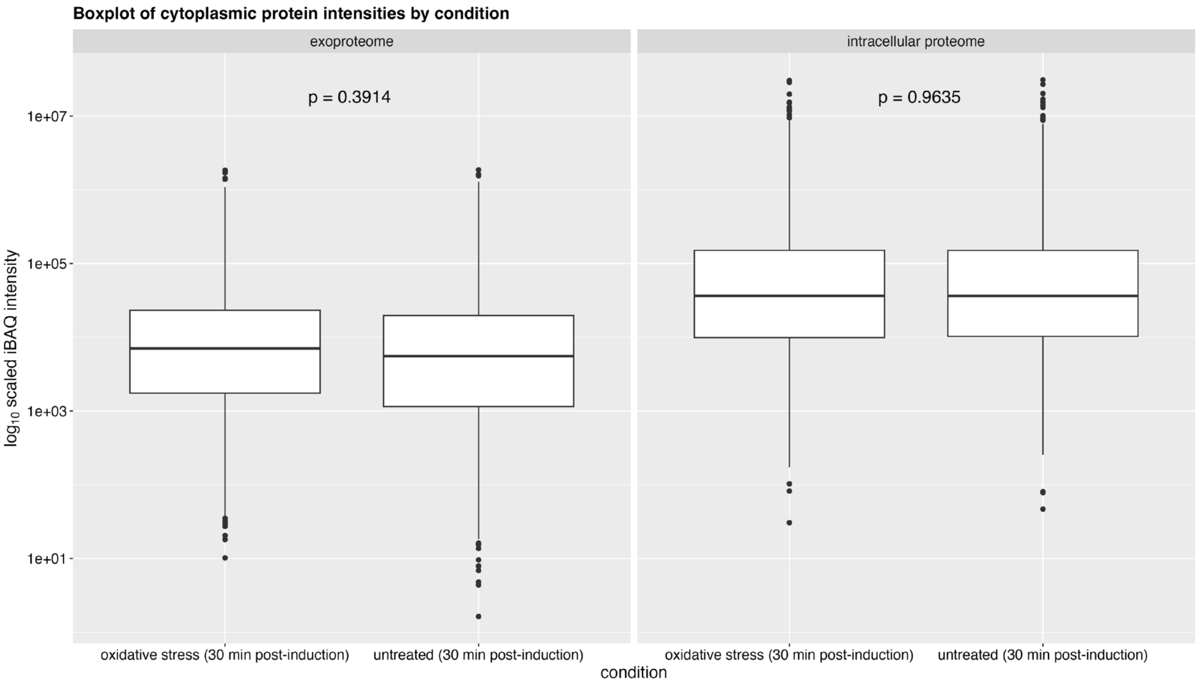

Figure S2 | Protein intensities of cytoplasmic proteins in the exoproteome and intracellular proteome. To assess the potential effect of lysis following oxidative stress, protein intensities in untreated and stressed conditions were compared. In the case of lysis, the intensities in the exoproteome should have increased after oxidative stress. However, the differences between conditions were not statistically significant, as determined by a t-test.

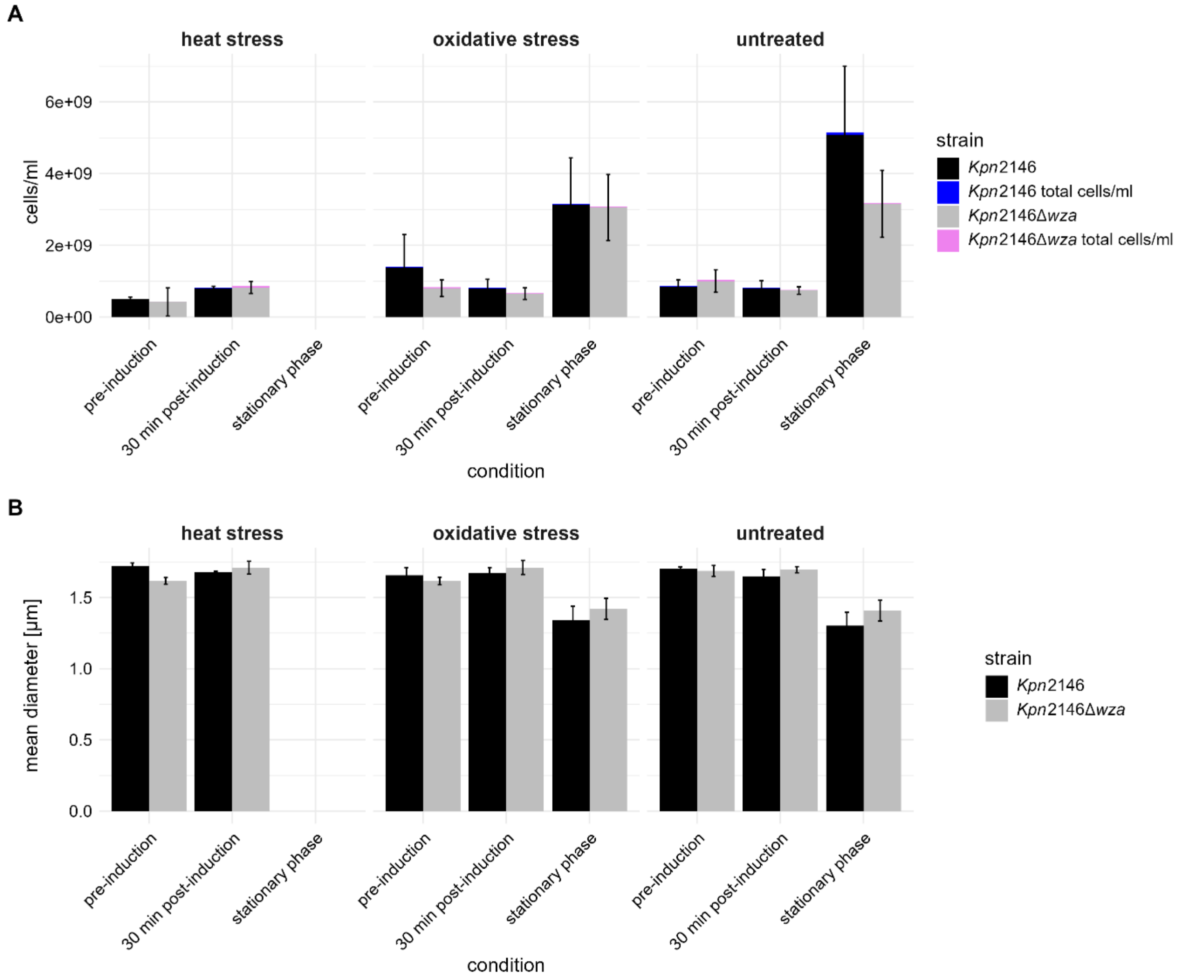

Figure S3 | Cell count and viability determination of the encapsulated *Kpn*2146 and capsule-deficient *Kpn*2146Δ*wza* strain. (A) The cells per ml were determined using the CASY® TT-2QA-2583. Black and grey bars represent the cells/ml, while the coloured bars highlight the total cells/ml. (B) The mean diameter of the measured bacterial cells was calculated and is depicted in micrometers (µm).

Supplementary Tables

Table A | Settings for reversed phase liquid chromatography (RPLC) and tandem mass spectrometry (MS/MS)

**Reversed phase liquid chromatography (RPLC)**	
Instrument	Ultimate 3000 RSLC (Thermo Scientific)
Trap column	75 µm inner diameter, packed with 3 µm C18 particles (Acclaim PepMap100, Thermo Scientific)
Analytical column	Accucore 150-C18, (Thermo Fisher Scientific)<br>25 cm x 75 µm, 2.6 µm C18 particles, 150 Å pore size
Buffer system	Binary buffer system consisting of 0.1% acetic acid in HPLC-grade water (solvent A) and 100% ACN in 0.1% acetic acid (solvent B)
Flow rate	300 nl/min
Gradient	Linear gradient of solvent B from 2% up to 25% for intracellular proteome analysis
	0min-2%<br>2min-5%<br>10min-7%<br>70min-25%<br>75min-40%<br>77min-90%<br>83min-90%<br>85min-2%<br>95min-2%<br>gradient of solvent B for exoproteome analysis
Gradient duration	120 min
Column oven temperature	40°C
**MS/MS Intracellular proteome**	
Instrument	Q Exactive HF mass spectrometer (Thermo Fisher Scientific)
Electrospray	Nanospray Flex™ Ion Source
Operation mode	Data-independent

**Full scan properties**	
MS scan resolution	60000
Normalized AGC target	5e6
Maximum ion injection time for the MS scan	200 ms
Scan range	333 to 1650 m/z
RF Lens	50 %
Spectra data type	Profile
**Dia properties (MS2)**	
Precursor mass range	333 to 1650 m/z
Resolution	30,000
Norm. MS/MS AGC target	3e6
Maximum ion injection time mode	Auto
Spectra data type	Profile
Microscans	1
Isolation window	56 windows, 13 m/z, 2 m/z overlap
Define first mass	200
Dissociation mode	Higher energy collisional dissociation (HCD)
Normalized collision energy	27.5 %
**MS/MS Exoproteome**	
Instrument	Orbitrap Exploris 480 mass spectrometer (Thermo Fisher Scientific)
Electrospray	Nanospray Flex™ Ion Source
Operation mode	Data-independent
**Full scan properties**	
MS scan resolution	120000
AGC target	3e6 (300%)

Maximum ion injection time for the MS scan	60 ms
Scan range	350 to 1200 m/z
Microscans	1
Polarity	Positive
RF lens	50%
Spectra data type	Profile
**Dia properties (MS2)**	
Resolution	30000
Maximum ion injection time for the MS/MS scans	Auto
Normalized AGC target	3e6
Spectra data type	Profile
Microscans	1
Isolation window	66
Isolation window width	13 m/z
Window overlay	2 m/z
Fixed first mass	200
HCD collision energy	30%

Table B | Spectronaut® parameters used for data analysis (DIA) of the intracellular proteome

Software	Spectronaut version 18.2 (Biognosys, Schlieren, Switzerland)
**Data extraction**	
**Intensity extraction MS1 and MS2**	maximum intensity
**MS1 and MS2 mass tolerance strategy**	dynamic
**XIC extraction**	
XIC extraction window	dynamic
**Calibration**	
Calibration mode	automatic
RT regression type	local (non-linear) regression
**Identification**	
Decoy method	mutated
Decoy limit strategy	dynamic
Machine learning	per run
Precursor Qvalue cutoff	0.001
Protein Qvalue cutoff	0.01
PValue Estimator	Kernel Density Estimator
**Quantification**	
Interference correction	True, MS1 Min. 2, MS2 Min. 3
Quantity MS level	MS2
Quantity type	area
Data filtering	Qvalue sparse
**Workflow**	
Profiling strategy	iRT profiling
Profiling Row Selection	Minimum Qvalue Row Selection, Qvalue Treshold 0.001
Profiling Target Selection	Profile only non-identified precursor,<br>Identification Criterion Qvalue, Treshold 0.001
Carry-over exact peak boundaries	False
Unify peptide peaks	Select corresponding peak

Table C | Spectronaut™ parameters used for data analysis (DIA) of the exoproteome

Software	Spectronaut version 18.6 (Biognosys, Schlieren, Switzerland)
**Data extraction**	
**Intensity extraction MS1 and MS2**	maximum intensity
**MS1 and MS2 mass tolerance strategy**	dynamic
**XIC extraction**	
XIC extraction window	dynamic
**Calibration**	
Calibration mode	automatic
RT regression type	local (non-linear) regression
**Identification**	
Decoy method	mutated
Decoy limit strategy	dynamic
Machine learning	per run
Precursor Qvalue cutoff	0.001
Protein Qvalue cutoff	0.01
PValue Estimator	Kernel Density Estimator
**Quantification**	
Interference correction	True, MS1 Min. 2, MS2 Min. 3
Quantity MS level	MS2
Quantity type	area
Cross-Run Normalization	True
Normalization Filter Type	Library (Bacillus_subtilis_N15_labeled)
Normalization Strategy	Local Normalization
Data filtering	Qvalue sparse
**Workflow**	
Profiling strategy	iRT profiling
Profiling Row Selection	Minimum Qvalue Row Selection, Qvalue Treshold 0.001
Profiling Target Selection	Profile only non-identified precursor, Identification Criterion Qvalue, Treshold 0.001

Carry-over exact peak boundaries	False
Unify peptide peaks	Select corresponding peak
**Spectral libraries**	
240216_Klebsiella_pneumniae_BAA2146_library_SI	
Precursors Targeted	61,348
Decoys added	6,116
Decoy method	mutated
Bacillus_subtilis_N15_labeled	
Precursors Targeted	21,727
Decoys added	5,000
Decoy method	mutated

Table D | Top ten ranked mean iBAQ protein intensities per condition of the intracellular proteome and exoproteome

Intracellular proteome

Protein ID (NCBI)	Protein	Description	Pathway(s)
AHI36588.1	Lpp[1-6]	Major outer membrane protein Lpp	NA
AHI33726.1	HupA[1-6]	DNA-binding protein HU-alpha	DNA Metabolism
AHI35335.2	OmpA[1-6]	Outer membrane protein A	Stress Response
AHI35569.1	GapA[1-6]	Glyceraldehyde-3-phosphate dehydrogenase A	Cofactors, Vitamins, Prosthetic Groups, Pigments; Carbohydrates; Stress Response
AHI34972.1	CspE[1, 3]	Cold shock-like protein CspE	Stress Response
AHI34235.1	Kpn2146_0778[1-5]	30S ribosomal protein S20	NA
AHI38371.1	RplP[1-6]	50S ribosomal protein L16	NA
AHI33881.1	GroS_1[2-6]	10 kDa chaperonin	Protein Metabolism
AHI33707.1	RplL[1, 3]	50S ribosomal protein L7/L12	NA
AHI37496.1	RpsP[1, 3-5]	30S ribosomal protein S16	Protein Metabolism
AHI35147.1	OmpX[1-6]	Outer membrane protein X	NA

AHI33882.1	GroL_1[2, 4]	60 kDa chaperonin	Protein Metabolism
AHI35067.1	GpmA[5, 6]	2,3-bisphosphoglycerate-dependent phosphoglycerate mutase	Carbohydrates
AHI34964.1	AhpC[6]	Alkyl hydroperoxide reductase subunit C	Sulfur Metabolism
AHI37277.1	Crr[6]	Glucose-specific phosphotransferase enzyme IIA component	Carbohydrates

[1]untreated condition 30 min after heat shock, [2]condition 30 min after heat shock, [3]untreated condition 30 min after oxidative stress, [4]condition 30 min after oxidative stress, [5]untreated condition in stationary phase, [6]condition in stationary phase after oxidative stress. NA: not significantly assigned.

**Exoproteome**			
**Protein ID (NCBI)**	**Protein**	**Description**	**Pathway(s)**
AHI35147.1	OmpX[1-6]	Outer membrane protein X	NA
AHI35335.2	OmpA[1-6]	Outer membrane protein A	Stress Response
AHI36588.1	Lpp[1-6]	Major outer membrane protein Lpp	NA
AHI33881.1	GroS_1[1-4]	10 kDa chaperonin	Protein Metabolism
AHI38385.1	RpsG[1-4]	30S ribosomal protein S7	Virulence; Protein Metabolism
AHI36355.1	Kpn2146_2954[1, 3-6]	SMP-30/Gluconolaconase/LRE-like region	NA
AHI33882.1	GroL_1[1, 2, 44]	60 kDa chaperonin	Protein Metabolism
AHI37718.1	Eno[2, 4]	Enolase	Carbohydrates

AHI33603.1	ZinT[1, 3, 5, 6]	Metal-binding protein ZinT	NA
AHI38375.1	RplB[2]	50S ribosomal protein L2	Carbohydrates
AHI34815.1	Kpn2146_1377[1-6]	Hypothetical protein	NA
AHI37616.1	OsmY_2[1, 3]	Osmotically-inducible protein Y	NA
AHI38371.1	RplP[2]	50S ribosomal protein L16	Virulence; Protein Metabolism
AHI37672.1	Kpn2146_4322[3, 5, 6]	Putative periplasmic iron-binding protein	NA
AHI38391.1	FkpA_2[5, 6]	FKBP-type peptidyl-prolyl cis-trans isomerase FkpA	NA
AHI35135.1	BhsA_1[5]	Multiple stress resistance protein BhsA	NA
AHI33526.1	DsbA_1[5]	Thiol:disulfide interchange protein DsbA	Protein Metabolism; Respiration
AHI33944.1	RpsR[4]	30S ribosomal protein S18	Protein Metabolism
AHI37865.1	Kpn2146_4523[6]	Uncharacterized lipoprotein YbaY/Fimbrial protein	NA
AHI36021.1	Kpn2146_2620[6]	Ambler Class A beta lactamase SHV-11	NA

[1]untreated condition 30 min after heat shock, [2]condition 30 min after heat shock, [3]untreated condition 30 min after oxidative stress induction, [4]condition 30 min after oxidative stress, [5]untreated condition in stationary phase, [6]condition in stationary phase after oxidative stress. NA: not significantly assigned.

Table E | Number of detected proteins (≥ 2 peptides) involved in *Kpn*2146 pathways assigned from the TheSEED database with corresponding proportions of the total assigned metabolic proteins

**Metabolic category**	**Intracellular proteins [n]**	**Proportion of metabolic proteins [%]**	**Exoproteome proteins [n]**	**Proportion of metabolic proteins [%]**
Amino Acids and Derivatives	191	11.6	167	11.1
Carbohydrates	254	15.5	238	15.8
Cell Division and Cell Cycle	19	1.2	19	1.3
Cell Wall and Capsule	106	6.5	103	6.9
Cofactors, Vitamins, Prosthetic Groups, Pigments	123	7.5	105	7.0
DNA Metabolism	90	5.5	83	5.5
Dormancy and Sporulation	3	0.2	3	0.2
Fatty Acids, Lipids, and Isoprenoids	48	2.9	41	2.7
Iron Acquisition and Metabolism	35	2.1	33	2.2
Membrane Transport	46	2.8	41	2.7
Metabolism of Aromatic Compounds	10	0.6	7	0.5
Miscellaneous	16	1.0	15	1.0
Motility and Chemotaxis	7	0.4	8	0.5
Nitrogen Metabolism	12	0.7	12	0.8

Nucleosides and Nucleotides	83	5.1	75	5.0
Phages, Prophages, Transposable elements	1	0.1	3	0.2
Phosphorus Metabolism	28	1.7	27	1.8
Potassium Metabolism	13	0.8	12	0.8
Protein Metabolism	124	7.6	121	8.1
Regulation and Cell Signaling	78	4.8	66	4.4
Respiration	53	3.2	46	3.1
RNA Metabolism	85	5.2	80	5.3
Secondary Metabolism	1	0.1	1	0.1
Stress Response	110	6.7	99	6.6
Sulfur Metabolism	38	2.3	37	2.5
Virulence	68	4.1	61	4.1
$\sum$	**1642**		**1503**	

Supplementary data

BLAST Alignment data, Supplementary data F

```
RID: 3CSPTCNK016 Job Title:Protein Sequence
Program: BLASTP
Query: unnamed protein product ID: lcl|Query_12293714(amino acid) Length: 202
Database: nr All non-redundant GenBank CDS translations+PDB+SwissProt+PIR+PRF
excluding environmental samples from WGS projects

Sequences producing significant alignments:
                                                                  Scientific
Common                    Max    Total Query   E     Per.   Acc.
Description                                                       Name
Name            Taxid      Score  Score cover Value  Ident  Len
Accession
type 3 fimbria major subunit MrkA [Gammaproteobacteria]
Gammaproteob... NA             1236       403    403   100%  1e-146 100.00
202        WP_002916128.1
type 3 fimbria minor subunit MrkF [Gammaproteobacteria]
Gammaproteob... NA             1236       153    153   93%   6e-48  43.43
211        WP_002916122.1

Alignments:

>MULTISPECIES: type 3 fimbria major subunit MrkA [Gammaproteobacteria]
Sequence ID: WP_002916128.1 Length: 202
Range 1: 1 to 202

Score:403 bits(1036), Expect:1e-146,
Method:Compositional matrix adjust.,
Identities:202/202(100%), Positives:202/202(100%), Gaps:0/202(0%)

Query  1    MKKVLLSAAMATAFFGMTAAHAADTTVGGGQVNFFGKVTDVSCTVSVNGQGSDANVYLSP  60
            MKKVLLSAAMATAFFGMTAAHAADTTVGGGQVNFFGKVTDVSCTVSVNGQGSDANVYLSP
Sbjct  1    MKKVLLSAAMATAFFGMTAAHAADTTVGGGQVNFFGKVTDVSCTVSVNGQGSDANVYLSP  60

Query  61   VTLTEVKAAAADTYLKPKSFTIDVSNCQAADGTKQDDVSKLGVNWTGGNLLAGATSKQQG  120
            VTLTEVKAAAADTYLKPKSFTIDVSNCQAADGTKQDDVSKLGVNWTGGNLLAGATSKQQG
Sbjct  61   VTLTEVKAAAADTYLKPKSFTIDVSNCQAADGTKQDDVSKLGVNWTGGNLLAGATSKQQG  120

Query  121  YLANTEASGAQNIQLVLSTDNATALTNKIIPGDSTQPKAKGDASAVADGARFTYYVGYAT  180
            YLANTEASGAQNIQLVLSTDNATALTNKIIPGDSTQPKAKGDASAVADGARFTYYVGYAT
Sbjct  121  YLANTEASGAQNIQLVLSTDNATALTNKIIPGDSTQPKAKGDASAVADGARFTYYVGYAT  180

Query  181  SAPTTVTTGVVNSYATYEITYQ  202
            SAPTTVTTGVVNSYATYEITYQ
Sbjct  181  SAPTTVTTGVVNSYATYEITYQ  202
```

```
>MULTISPECIES: type 3 fimbria minor subunit MrkF [Gammaproteobacteria]
Sequence ID: WP_002916122.1 Length: 211
Range 1: 14 to 210

Score:153 bits(387), Expect:6e-48,
Method:Compositional matrix adjust.,
Identities:86/198(43%), Positives:122/198(61%), Gaps:11/198(5%)

Query  14   FFGMTAAHAA---DTTVGGGQVNFFGKVTDVSCTVSVNG--QGSDANVYLSPVTLTEVKA  68
            F G  AA +A   +T    G+V+F G+VTD+SC+V++NG       NV+L+PV+L EV
Sbjct  14   FCGSLAAPSAWGFETNYDRGRVDFAGRVTDISCSVALNGGQHAGSGNVWLAPVSLAEVHD  73

Query  69   AAADTYLKPKSFTIDVSNCQ-AADG--TKQDDVSKLGVNWTGGNLLAGATSKQQGYLANT  125
              A  ++KP+ FT+ +SNCQ   DG    QD+V ++ V W  G LL    ++  GYLANT
Sbjct  74   RGAGAFMKPQPFTLALSNCQLRHDGGAASQDEVRRVSVRWVDGFLLTAVGNENAGYLANT  133

Query  126  EASGAQNIQLVLSTDNATAL--TNKIIPGDSTQPKAKGDASAVADGARFTYYVGYATSAP  183
               GAQNI L LST++   L  +NKI+P D  Q + +   SAV+ G  FTYYVGY +  P
Sbjct  134  LPDGAQNIYLALSTNDNNTLDKSNKIVPADPQQNQVRLQESAVS-GGLFTYYVGYVSPTP  192

Query  184  TTVTTGVVNSYATYEITY  201
             + T+G + S+AT+E+ Y
Sbjct  193  KSATSGPITSWATWELVY  210
```
